# The Effect of Mushroom Beta-Glucans from Solid Culture of *Ganoderma lucidum* on Inhibition of the Primary Tumor Metastasis

**DOI:** 10.1155/2014/252171

**Published:** 2014-04-01

**Authors:** Shiu-Nan Chen, Ching-Sheng Chang, Ming-Hsin Hung, Sherwin Chen, William Wang, Cheng-Jeng Tai, Chung-Lun Lu

**Affiliations:** ^1^College of Life Science, National Taiwan University, Section 4, Roosevelt Road, Taipei 10617, Taiwan; ^2^Department of Research and Development, Super Beta Glucan Inc., Irvine, CA, USA; ^3^Department of Internal Medicine, School of Medicine, College of Medicine, Taipei Medical University, Taipei 110, Taiwan; ^4^Division of Hematology and Oncology, Department of Internal Medicine, Taipei Medical University Hospital, Taipei 110, Taiwan

## Abstract

This study evaluates the effect of mushroom beta-glucans (MBGS) derived from solid culture of *Ganoderma lucidum* on tumor inhibition by examining size of the primary tumor and rate of metastasis in Lewis lung carcinoma (LLC) bearing mice (C57BL/6), given oral administration of MBGS with radiation therapy. A previous result showed that MBGS enhances NK cell-mediated cytotoxicity in mice without LLC bearing in advance. Furthermore, applications of MBGS in conjunction with radiation therapy were effective in controlling tumor growth, and rate of metastasis, life threatening, and can potentially serve as a protective factor for wounds and hair loss that resulted from the overgrowth of primary tumor in LLC bearing mice.

## 1. Introduction

Mushrooms have been valued for their health benefits and medicinal effects for centuries. One of the special components found from mushrooms is beta-glucan, which is predominantly composed in the fungal cell wall and is mostly composed of beta-D-glucose. In many researches, beta-glucan effectively stimulates the host immune response to defend against bacterial, viral, fungal, or parasitic infections [1]. Moreover, it is known as biological response modifier since it primarily achieves its disease protective activity through modulating the host immune system [2]. The stimulation of beta-glucan to macrophages, neutrophils, and natural killer (NK) cells is proved by binding to the receptor (dectin-1) of these cells and modulates the systems [3, 4]. In clinical applications, beta-glucan is usually used as an adjuvant to enhance the effectiveness of the medicine [5, 6]. To sum up the experimental and clinical results, the potential anticancer activity from beta-glucan has been proven, and thus beta-glucan has been gaining prominence in clinical research during the past few years [6, 7].

The rapidly grown cancer cells turn into tumors, which compete with other somatic cells for space and nutrient. The tumor oppresses normal tissues, affects the normal function of surrounding tissues, and invades adjacent blood vessels or the lymphatic system which leads to metastasis [8]. In fact, many cancer patients do not decease from the exacerbation of the primary tumors. Instead the most common cause of death is from the establishment of secondary tumors in other areas through metastasis. When these tumor cells successfully proliferate in the new host environment, a secondary tumor is formed, which completes the metastatic process and is a potential risk factor during current cancer therapy and patients life threaten [9].

Natural killer (NK) cells have the ability to distinguish self- versus non-self-cells through the MHC- (major histocompatibility complex-) class I molecules on the cell surface [10]. The MHC-class I molecules on self-cells inhibit the NK cell-mediated cytotoxicity. Atypical cells or infected cells will try to evade being identified by the host immune system through reducing or eliminating the cell surface presentation of MHC-class I molecules. Since most cancer cells are derived from the abnormal proliferation of self-cells, a normal immune system will not necessarily distinguish and eradicate the cancer cells effectively. Therefore the addition of cytotoxic function, such as NK cell-mediated cytotoxicity in eliminating cancer cells, plays an important role in cancer therapy [11].

This study estimated the NK cell-mediated cytotoxicity of mice which is treated by MBGS and, furthermore, used the tumor-bearing murine model of inducing the metastasis from the primary tumor by radiation [12] and observes for the effectiveness of MBGS in conjunction with the radiation therapy to control cancer metastasis.

## 2. Materials and Methods

### 2.1. Mushroom Beta-Glucans (MBGS) Preparation and Cell Culture

Manufacturing process of MBGS was initiated by culturing of* G. lucidum* in a culture broth containing glucose, lactose, galactase, sucrose, mannose, and yeast extract using a shaker incubator in temperature that ranged from 21 to 25°C for 2 weeks. Subsequently, cultured mycelium of* G. lucidum* was then inoculated into a sterile solid medium containing brown rice, oats, and buckwheat in a temperature of 25°C for approximately 6 months. Following emergence of the fruit body, all materials in the culture flasks were then dried and grinded into a fine powder. The powder was then dissolved in distilled water at 1 : 5 ratio and stirred using a magnetic stirrer for 6~10 h at 20~30°C. Following centrifugation, 95% of alcohol was then added into the supernatant to give a final concentration of 60% alcohol. The precipitation was then collected and redissolved in approximately 3 times of the distilled water. The crude MBGS solution was then concentrated by a ceramic membrane. HPLC analysis showed that MBGS contained high molecular weight particles that ranged from 9.6~298 kDa, and GC-MS analysis showed that MBGS contained 2-; 4-; and 6-linked galactopyranosyl residues and 3-; 4-; 3,4-; 2,4-; 4,6-; and 3,4,6-linked glucopyranosyl residues. The crude MBGS solution was dried and grinded into the fine powder form. Beta-glucan concentration of MBGS determination by commercial “Megazyme (Ireland) mushroom and yeast beta-glucan kit” was demonstrated at approximately 70–75%.

Lewis lung carcinoma (LLC) cell line and YAC-1 cell lines were purchased from the Bioresource Collection and Research Center (Taiwan). The LLC cells were anchorage-dependent which were cultured in DEME medium (with 10% FBS). The YAC-1 were suspension cells and being cultured in PRMI-1640 medium (with 10% FBS). When the cells grew to the designated quantity, they were collected separately and used as tumor inducing cells for the mice and as target cells for the cytotoxicity analysis.

### 2.2. Dosage Determination

Since MBGS is a novel, highly purified beta-glucan that has been utilized in the present study, the dosages for mice were determined as 10 mg/kg/day based on a prior study conducted by Itoh et al., which utilized a similar polysaccharide isolated from* Agaricus blazei* with antitumor properties [13].

### 2.3. Experiment Animals and Tumor Bearing Procedures

C57BL/6 mice (6 weeks old) were purchased from the Laboratory Animal Center in National Taiwan University College of Medicine and housed in animal rooms in compliance with the institutional guidelines. For the present study, animals with the same gender and treatments were housed together by using polycarbonate cages with paddy husk bedding in the animal room. Rodent feed 5010, LabDiet, PMI, Nutrition International (Brentwood, MO) and drinking water, was provided ad libitum throughout the study period. The room temperature and relative humidity were maintained at 21 ± 2°C and 55 ± 20%, respectively, with a 12 h light/dark cycle. The animals were allowed to acclimatize for a minimum of 6 days before the initiation of experiments.

The LLC cells cultured with a preset length of time were treated with trypsin and then brought into suspension and collected by centrifugation. The cells collected were rinsed and resuspended with PBS to undergo further centrifugation for cell collections. This process was repeated for 3 times. Cells collected during the final round of centrifugation were brought into suspension in PBS, counted, and diluted to a cell density of 2 × 10^5^/*μ*L. Using a procedure modified from Camphausen et al. [12] and Gorelik et al. [14], 50 *μ*L of LLC cell fluids were injected subcutaneously into the right hind leg of the mice and monitored for 3 to 5 days to ensure the implantation was successful.

### 2.4. The Effect of MBGS on NK Cell-Mediated Cytotoxicity in Mice

To observe the change in cytotoxicity associated with the different lengths in MBGS treatments, a total of 27 C57BL/6 mice (6 weeks old) were randomly divided into 9 groups. With the control group being fed standard diets, the other 8 groups received MBGS (gavage) treatment between 1 and 8 days. Mice were euthanized and the monocytes from the spleen were extracted for measurements of cytotoxicity. During the extraction procedure, spleens were removed and shredded with forceps, followed by separating the monocytes with centrifugation using Histopaque. The isolated monocytes were used as the effector cells after being washed twice with PBS buffer and had the cell density adjusted to 1 × 10^6^/mL in RPMI 1640 medium.

YAC-1 cells, intended to be used as the target cells, were collected by centrifugation and had the cell density adjusted to 1 × 10^6^/mL. The cells were then stained with DiOC-18 at 37°C, 5% CO_2_ for 20 min, followed by a PBS rinse, and suspended to 1 × 10^6^/mL in RPMI 1640 medium. For the assay of NK cell-mediated cytotoxicity, the effector and target cells were mixed in ratios of 10 : 1 followed by adding the propidium iodide (PI) staining solution to each mixture. Finally, the cell mixtures were incubated at 37°C, 5% CO_2_ for 2 h, and analyzed with flow cytometer. Lysed (PI^+^ and DiOC-18^+^) and viable (DiOC-18^+^ and PI^−^) YAC-1 cells were identified by their dual- or single-positive staining. Assessment of the NK cell-mediated cytotoxicity was defined by the percentage increase in cytotoxicity relative to the baseline level set by the control group (100%).

### 2.5. Tumor-Bearing Mice and Radiation Treatment Procedure

40 tumor-bearing mice were randomly divided into 4 groups: the control group, the group assigned to MBGS treatments only, the group assigned to radiation treatments only, and the group assigned to both MBGS and radiation treatments. To set up for the radiation treatments, LLC tumor-bearing mice were immobilized in a customized harness that allowed the right hind leg to be exposed. The tumor-bearing sites were irradiated by Cobalt 60 unit with 10 Gy per day for 8 consecutive days. MBGS treatments were provided for 22 days throughout the intervention period, which started out from initiation of the radiation therapy and continued for another 14 days after that. At the 23rd day following the interventions, 5 mice from each group were euthanized immediately to measure the primary tumor sizes. Moreover, the numbers of tumor nodules in the lung were counted to analyze the extent of metastasis. Finally, the remainders of the mice were observed daily and euthanized when criteria from the guidelines for euthanasia [15] were met and the length of survival was recorded.

### 2.6. Statistical Analysis

One-way ANOVA followed by Turkey's HSD test was used to evaluate the statistical significances of differences amongst groups. A *P* value less than 0.05 (*P* < 0.05) was considered of a statistical significance. Results are presented as mean ± SD.

## 3. Results

### 3.1. The Effect of MBGS on NK Cell-Mediated Cytotoxicity in Mice

**Figure 1:**
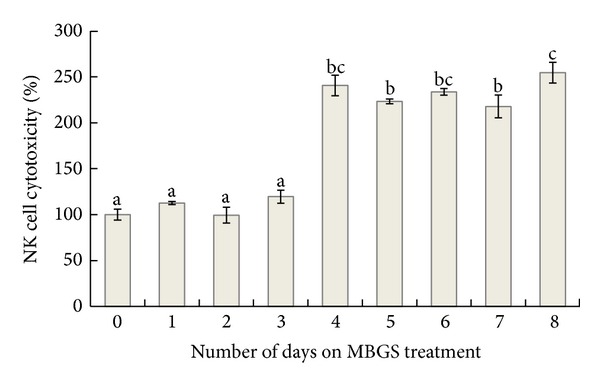
The effect of MBGS on NK cell-mediated cytotoxicity. Changes in cytotoxicity upon MBGS treatments were recorded daily. The cytotoxicity of the group that did not receive MBGS treatments (control) was assigned to 100%, and the relative cytotoxicity level after taking MBGS treatments was calculated. Each histogram represented the mean ± SD cytotoxicity measured from 3 mice. The lower case letters indicate significant difference between groups from each day (*P* < 0.05).

The NK cell-mediated cytotoxicity from healthy C57BL/6 mice receiving MBGS treatments was recorded for 8 consecutive days. The cytotoxicity from the control group that did not receive MBGS treatments was referenced as 100%. Results indicated that the cytotoxicity level increased significantly upon MBGS for 4 consecutive days, and with the level maintained above the 200% mark (Figure 1).

### 3.2. The Effects of Administering MBGS on Cancer Therapy

**Figure 2:**
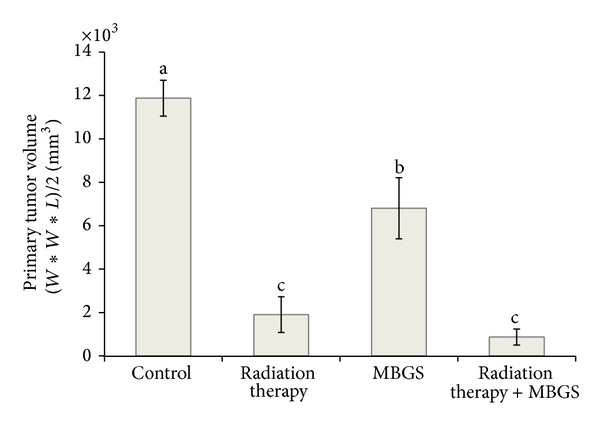
Different treatments options versus primary tumor volume. The primary tumor volume was the greatest in the control group as compared to the others. The group receiving radiation therapy only and the one receiving both radiation therapy and MBGS treatments showed statistically significant reductions in the primary tumor volume. Each histogram represents the mean ± SD of primary tumor volumes calculated from each group containing 5 mice. The lower case letters indicate significant difference between groups (*P* < 0.05).

**Figure 3:**
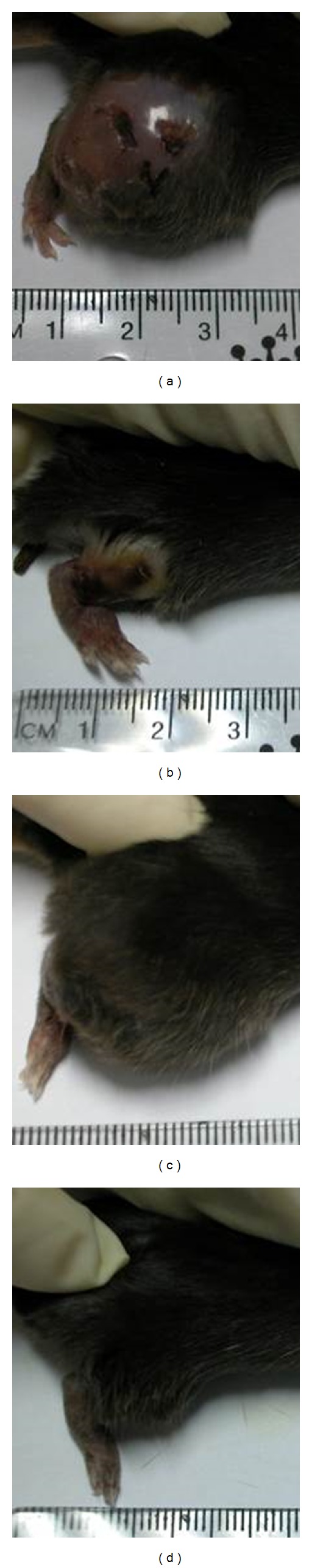
Observation on the tumor-bearing site of experimental mice. The mouse from the control group showed the greatest primary tumor volume and demonstrated external wounds and hair loss that resulted from the rapid growth of the primary tumor (a). The mouse from the radiation therapy group showed reduction in primary tumor size but with wounds eschar on the surface of the hind leg (b). Both groups receiving MBGS treatments had improved condition in hair loss and wounds regardless of whether the radiation therapy was provided ((c) and (d)).

To estimate the tumor growth phenomenon, the primary tumor was calculated with an ellipsoid volume calculator and the volume average was recorded from each group. The volume average of the primary tumor was the greatest (11876 mm^2^) in the control group (Figure 2 and Figure 3(a)). The group assigned to radiation therapy only had significant primary tumor growth inhibition rates when compared to the control group; however, the mice from this particular group also had significant hair loss and external wounds on the surface of the hind leg (Figure 2 and Figure 3(b)). Both groups that received MBGS treatments had a significant decrease in the primary tumor volume than control (Figure 2). In addition, the less hair loss and less severe wounds occurred in groups that received MBGS treatments (Figures 3(c) and 3(d)).

**Figure 4:**
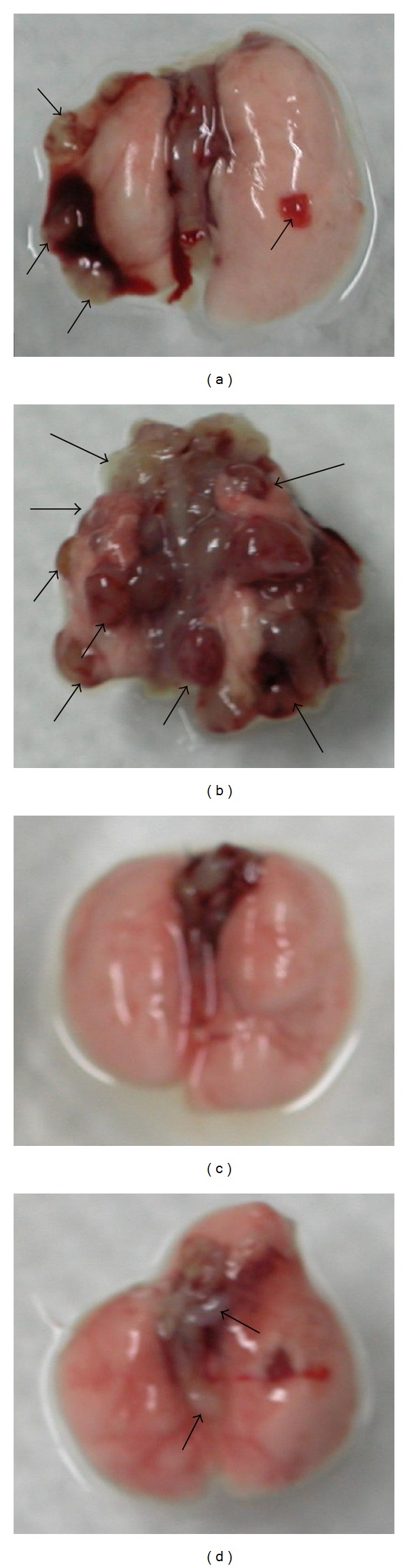
Necropsy examinations of mice lungs. Lungs from the tumor-bearing mice were removed and visually examined for the numbers of metastatic tumor nodules. As the results indicated (a) in the control group lungs with a few surface metastatic tumors nodules (arrows); (b) in the radiation therapy group lungs with a large number of surface metastatic tumors nodules (arrows); (c) in the MBGS treatments group lungs appeared to be free of metastatic tumor nodules; (d) in the combination of MBGS treatments and radiation therapy group lungs with a few surface metastatic tumors nodules (arrows).

**Figure 5:**
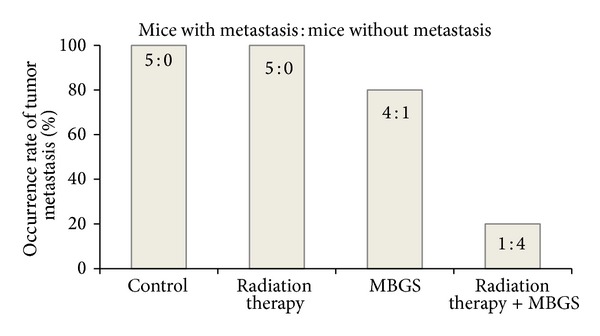
Different treatments options versus the incidence of metastasis. The incidence of metastasis was the greatest in the control and radiation therapy group (100%), followed by the group receiving MBGS treatments (80%) and the combination of MBGS treatment and radiation therapy group (20%).

**Figure 6:**
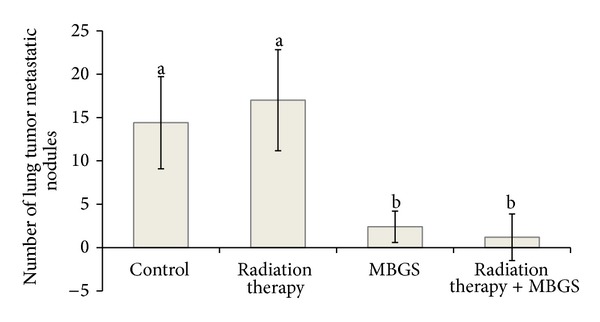
Comparisons in the number of metastatic nodules. At the 23rd day following the interventions, mice from each group were euthanized and the numbers of tumor nodules in the lung were counted to analyze the extent of metastasis. Both groups received MBGS treatments had significant lower findings in tumor metastatic nodules than the ones without (*P* < 0.05). Each histogram represented the mean ± SD nodules found in 5 mice. The lower case letters indicate significant difference between each group (*N* = 5) (*P* < 0.05).

Incidence of metastasis was calculated by recording the numbers of mice with metastasis versus the ones without at the end of the experiment. The metastatic tumor nodules visual examination was shown in Figure 4. Mice without metastasis were confirmed by visual confirmation and histopathological analysis. As indicated, the metastasis incidence was the greatest in the control and radiation therapy group (100%), followed by the group receiving MBGS treatments (80%) and the combination of MBGS treatments and radiation therapy group (20%) (Figure 5). Finally, the number of metastatic tumor nodules from each treatments group was counted and calculated for the average (as shown in Figure 6). The results indicated that mice from the radiation therapy group had the highest numbers of metastatic tumor nodules (nodules = 17), followed by the control group (nodules = 14), MBGS treatments group (nodules = 2), and the MBGS and radiation therapy group (nodules = 1). In comparison, both groups that received MBGS treatments had significant lower findings in tumor metastatic nodules than the ones without (*P* < 0.05) (Figure 6).

**Figure 7:**
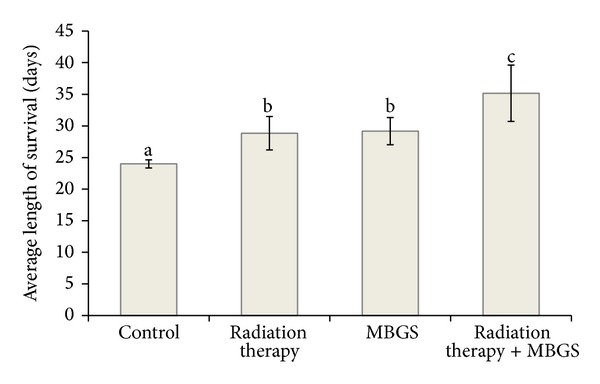
Comparison in the average length of survival. The average length of survival in group receiving both MBGS treatments and radiation therapy is 35 days, followed by the MBGS only treatments group (29 days), and the radiation only therapy group (28 days) compared to the control group (24 days), respectively. The lower case letters indicate significant difference between each group (*N* = 5) (*P* < 0.05).

The length of survival was recorded from each group and calculated for the average length of survival. Results showed a significant longer average length in group receiving both MBGS treatments and radiation therapy (35 days) (*P* < 0.05), followed by the MBGS only treatments group (29 days) (*P* < 0.05), and the radiation therapy group (28 days) (*P* < 0.05) compared to the control group (24 days), respectively (Figure 7).

## 4. Discussion

Beta-glucan was known to enhance and stimulate the immune system and express antitumoral activities in animal models [3, 4, 16]. Compared to the other beta-glucans extracted from the yeast or oats that promoted similar antimetastatic activities, beta-glucans from the mushroom were comprised of short *β* (1, 6) branches coming off a *β* (1, 3) backbone, thereby lacking the extra *β* (1, 3) branch extending from the *β* (1, 6) branch point [17, 18]. In addition to the structural difference, chemical viabilities including molecular weight and water solubility also made the beta-glucans from mushrooms more bioavailable and favorable for adjunct therapeutic applications [19–22].

NK cells are specialized large granular lymphocytes of the innate immune system responsible for eliminating virus-infected and tumor cells [23]. Also, these cells triggered spontaneous cytotoxicity that plays a critical role in immune surveillance and cancer therapy [24]. In 2000, research indicated that the cytotoxic anticancer agents have immunomodulating effects, which has been shown to enhance the activation of macrophages with associated increases of cytokines, can exert immunity-dependent curative effects in mouse tumor models [25]. In the present study, the potential adjunct therapeutic effects of MBGS treatment in a murine tumor-bearing model have been observed. The antimetastatic effect, as demonstrated in the present study, might be a result of an increase in NK-cell mediated cytotoxicity, as previously suggested by Di Luzio and Williams [26]; Ŝandula et al. [27]; and Chen et al., 2011 [17]. The NK cell-mediated cytotoxicity increases significantly after four days of MBGS treatment in mice. Additionally, a continuous daily MBGS treatment effectively maintains NK cell-mediated cytotoxicity. Despite the lack of statistical significant difference in primary tumor size between the group that received a combination of MBGS and radiation therapy and the group that received only the radiation therapy, the combination treatment group demonstrates a promising result for the overall treatment performance when factors such as damages inflicted on the outer epithelial layer, rate of tumor metastasis, and the average length of survival are compared in this study.

In 1981, effective results of single injection of 100 or 200 mg/kg or daily injections of 20 or 50 mg/kg of schizophyllan after removal of the primary tumor markedly inhibited pulmonary metastases [28]. As in the murine tumor-bearing model, the incidence of metastasis was 100% in both the control and radiation therapy group, 80% in the group receiving MBGS treatment and 20% in the group receiving both MBGS treatment and radiation therapy. These results suggest that the glucans, such as MBGS acts as an effective adjunct therapy to radiation therapy in reducing metastatic potential of the primary tumor. This is further confirmed during the necropsy by the large numbers of metastatic tumor nodules found in the lung tissues from both groups that did not receive MBGS treatments. We believe that the increase of NK cell-mediated cytotoxicity is highly associated with the protective effect in reducing the incidence of metastasis.

As another marker for the treatment efficacy, we observed an increasing trend in the length of survival in the treatment groups, with the control group being the shortest (24 days) and the group receiving a combination of MBGS treatment and radiation therapy being the longest (35 days). Moreover, the group that received the combination treatments survived 7 additional days than those who received radiation therapy only (28 days), suggesting an improved posttreatment prognosis using MBGS as an adjunct therapeutic agent with radiation therapy to increase the length of survival.

Furthermore, aside from lethargy, fatigue, and unintentional weight loss, which are common clinical manifestations for the cancer bearing mice, we have also observed open wounds and severe alopecia inflicted by the rapid growth of the primary tumor, from which the symptoms cannot be alleviated by the radiation therapy. However, within groups that received MBGS treatments, there are minimal hair loss and less severe wounds observed on the surface of the primary tumor. Findings from Kougias et al. indicate that in addition to macrophages, neutrophils and NK cells, membrane receptors for beta-glucan, are present in the human dermal fibroblasts [29], which promote wound repair and re-epithelialization of a full-thickness skin by stimulating human dermal fibroblast collagen biosynthesis through a nuclear factor-1 dependent mechanism [30, 31]. In addition, as suggested by prior researches, interferon-*γ* (IFN-*γ*), one of the primary cytokine produced by NK cells responsible for cancer immunosurveillance and immunoediting, can effectively active macrophage and dendritic cell to promote immunotherapeutic approaches to control and/or eliminate cancers [32]. As demonstrated from the result of this study, we suggested that the inhibition of the primary tumor metastasis was related to the expression of IFN-*γ*. This hypothesis was further confirmed by an unpublished data from a follow-up study which has shown a comparable outcome with respect to the current one.

Radiation therapy is one of the mainstays in cancer therapies to date. During the treatments process, high energy waves are projected to the cancerous growth, causing damage within the cells and ultimately cause the tumor to shrink. However, one of the disadvantages of radiation therapy is that the effectiveness is limited only to the localized tumor rather than a metastatic cancer, and a combination of immunotherapy and radiation therapy is recommended for the best posttreatments prognosis [33]. In this study, we look at the potential mechanism of MBGS treatments in reducing the rate of tumor growth and enhancing NK cell-mediated cytotoxicity against LLC tumor in mice. In addition, the results have further suggested that MBGS treatments reduce the rate of metastasis and improve the effectiveness of radiation therapy by providing additional length of survival for the tumor-bearing mice.
